# Creating a network of importance: The particular effects of self-relevance on stimulus processing

**DOI:** 10.3758/s13414-020-02070-7

**Published:** 2020-06-17

**Authors:** Sarah Schäfer, Dirk Wentura, Christian Frings

**Affiliations:** 1 Cognitive Psychology, University of Trier, D-54286 Trier, Germany; 2 General Psychology and Statistics, Saarland University, Saarbrücken, Germany

**Keywords:** Attention: Selective, Visual perception, Perceptual categorization and identification

## Abstract

Several factors guide our attention and the way we process our surroundings. In that regard, there is an ongoing debate about the way we are influenced by stimuli that have a particular self-relevance for us. Recent findings suggest that self-relevance does not always capture our attention automatically. Instead, an interpretation of the literature might be that self-relevance serves as an associative advantage facilitating the integration of relevant stimuli into the self-concept. We compared the effect of self-relevant stimuli with the effect of negative stimuli in three tasks measuring different aspects of cognitive processing. We found a first dissociation suggesting that negative valence attracts attention while self-relevance does not, a second dissociation suggesting that self-relevance influences stimulus processing beyond attention-grabbing mechanisms and in the form of an “associative glue,” while negative valence does not, and, last but not least, a third dissociation suggesting that self-relevance influences stimulus processing at a later stage than negative valence does.

## Introduction

Considering the vast amount of information flooding our senses in every waking moment and the way our cognitive system selects only a small amount of that information for further processing, one gains the impression that we are guided through a labyrinth of alternatives. A main organization principle of the way stimuli influence cognition and behavior, is whether they guide our attention automatically or whether attention is voluntarily allocated to them. In the case of attentional capture, empirical evidence strongly supports differentiation between the processing of stimuli that attract attention automatically, and those to which attention is allocated voluntarily. *Automatic* attentional capture is defined as involuntary and independent of the fact of whether attending to the stimulus is helpful or distracting; some speak of a bottom-up or stimulus-driven attention allocation in this case. *Voluntary* attentional capture, in contrast to that, is supposed to be a goal-directed, controlled, top-down way of allocating our attention (for a comparison of both, see, e.g., Yantis, 1993).

### The effects of self-relevance: are they purely attention-grabbing?

Self-relevance has been discussed as a clear candidate in the debate about which stimulus characteristics guide our attention automatically. Task-irrelevant, self-related stimuli have been shown to impede performance in different selective-attention paradigms. For example, the participant’s face as flankers in a name-identification task resulted in higher reaction times compared to a neutral face (Brédart et al., 2006), the cuing effect in a visual-search task was larger with the participant’s own name than with someone else’s name, and in an anti-saccade task, participants responded slower when they had to prevent their attention from being captured by their own name than by a neutral name (Alexopoulos et al., 2012; for supporting findings with psychophysiological measures, see also Gray et al., 2004).

Yet, recently, using a new task to measure self-prioritization (Sui et al., 2012), it has been found that self-relevance facilitates processing when it is task-relevant, but the very same stimuli did not elicit prioritized processing in a purely perceptual task (Falbén et al., 2019). This finding suggests that the effects of self-relevance on cognitive processing are context or task dependent. In the same vein, self-relevance has been described as acting as a “golden thread” in connecting stimuli (or brain regions processing self-relevant stimuli; Sui, 2016). According to this, self-relevance is not assumed to automatically allocate attention, but to influence cognition and/or behavior via advantages in associative learning (Sui, 2016). These interpretations challenge the way in which we typically think about effects of self-relevance. First, self-relevant stimuli may not automatically allocate attention, but are rather processed differently (for a related argument, see Frings, 2006). Second, self-relevance guides behavior by the facilitation of associative learning.

Like self-relevant stimuli, other stimuli, which are known to allocate attention automatically, are negative, threatening stimuli. For example, negative schematic faces revealed an interference effect when presented as distractors flanking a target (Barratt & Bundesen, 2012). Moreover, empirical evidence suggests an advantage in accessing awareness (Stein & Sterzer, 2012) as well as a delayed disengagement (Müller et al., 2016) for negative stimuli in comparison to neutral stimuli. Several studies reported delayed responses due to negative valence in the emotional Stroop task (see, e.g., Frings et al., 2010; Kahan & Hely, 2008; McKenna & Sharma, 2004; Pratto & John, 1991; Wentura et al., 2000), a variant of the color-word Stroop task (Stroop, 1935). Note that there is a debate on whether positive stimuli attract attention as well (e.g., Anderson et al., 2013; Brosch et al., 2008; Müller et al., 2016; Wentura et al., 2000, 2014). Especially in the emotional Stroop task, several studies failed to find an attention-grabbing effect of positive stimuli while they found an effect of negative stimuli (see, e.g., Bertels & Kolinsky, 2015; Kahan & Hely, 2008; but see Wentura et al., 2000, for a special type of positive stimuli). However, this debate is only of marginal importance in the present article.

More important in the present context is that until now it has been unclear whether negative stimuli might facilitate associative learning (in the sense of a prioritization effect) as well. We know of no study directly targeting this issue. Studies by MacKay and colleagues (MacKay et al., 2004; MacKay & Ahmetzanov, 2005) come closest to this issue. The authors argue that the binding of negative words’ meaning to salient contextual aspects is facilitated. However, their experiments tested for long-term memory effects of a special type of negative words (taboo words) and not for the kind of prioritization effect as found for self-relevant stimuli. Thus, as a leading hypothesis, we propose that prioritization as assessed by the matching paradigm is special for self-relevant items.

Taken together, if there is more to self-relevance than automatic attention allocation, more specifically, if self-relevance serves as an *associative glue* (e.g., Sui 2016), meaning that it facilitates associations between stimuli, then a different pattern of results should be observable for negative and self-relevant stimuli in tasks measuring different aspects of cognitive processing.

#### Study overview

In three experiments we compared the processing of self-relevant and negative stimuli. First, we used the emotional Stroop paradigm, which is by and large considered to measure automatic attentional capture of task-irrelevant stimulus characteristics (Experiment 1). Here, while participants are instructed to classify the color of visually presented words on the screen, responses on negative words are slowed down compared to responses on neutral words (for a review, see Williams et al., 1996; for a meta-analysis, see Phaf & Kan, 2007). While the emotional Stroop effect has been assumed previously to represent a generic slow-down, comparable to a freezing response, meanwhile the effect of (task-irrelevant) negative valence is discussed in depicting a more long-lasting attentional effect due to a delay in the attentional disengagement from negative stimuli than from neutral stimuli (Bertels & Kolinsky, 2015; Estes & Verges, 2008). Hence, we hypothesize a significant interference effect of negative stimuli in Experiment 1, whereas our hypothesis postulates a significantly smaller or even non-significant effect of self-relevance in this paradigm.

**Fig. 1:**
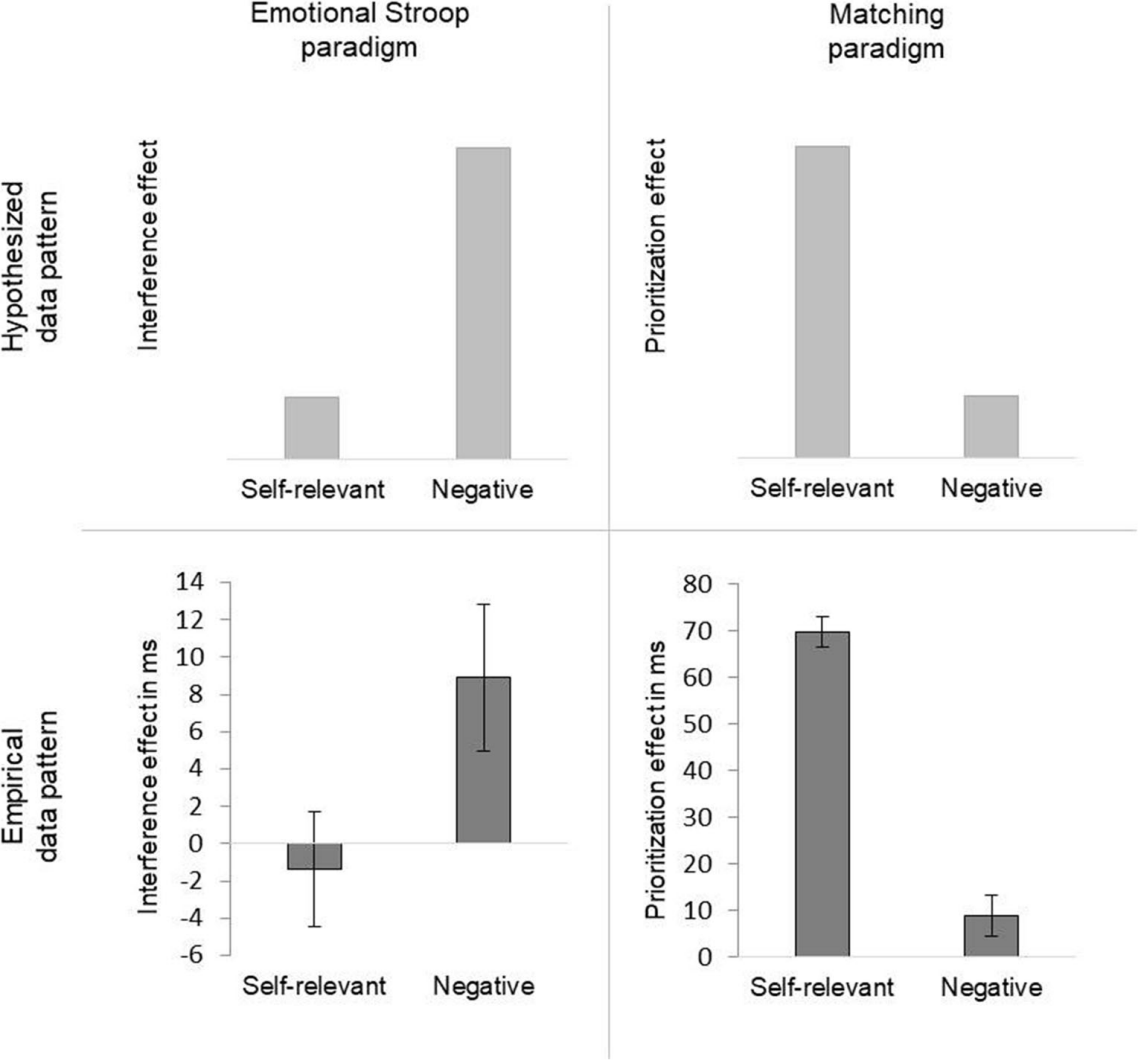
Hypothesized (upper row) and empirical (lower row) data pattern in the emotional Stroop paradigm (Exp. 1) and the matching paradigm (Exp. 2). Interference effects for self-relevant and negative words in the emotional Stroop paradigm are depicted as differences in reaction times (RTs) between the self-relevant and neutral condition or the negative and neutral condition. Prioritization effects in the matching paradigm for self-relevant as well as negative associations are depicted as differences in RTs between the self-relevant and neutral condition or the negative and neutral condition. Error bars indicate standard errors of the mean

Second, we used the matching paradigm as introduced by Sui et al. (2012) in Experiment 2. In this paradigm, formerly neutral stimuli are associated with the self and, subsequently, a robust prioritization (faster and more accurate responses) of these newly acquired self-associations is observed, the so-called self-prioritization effect (SPE; see, e.g., Mattan et al., 2014; Schäfer et al., 2015; Sui et al., 2014). However, different studies on the SPE suggested different underlying processes of self-relevance effects. A clear understanding how self-relevance guides cognitive processes in this task is still to be made (for evidence for a learning advantage of self-associated material, see Fuentes et al., 2016; for evidence for a perceptual advantage of self-associated material, see Macrae et al., 2017; for a review, see Sui & Humphreys, 2015b). Hence, we hypothesize a significant prioritization effect for self-relevant stimuli in Experiment 2, whereas our hypothesis postulates a significantly smaller and non-significant effect of negative valence in this paradigm. See Fig. 1 for the hypothesized data pattern for self-relevant and negative stimuli in Experiments 1 and 2.

Third, in order to compare the effects of self-relevance and negative valence on stages of stimulus processing, we conducted a third experiment using the psychological-refractory-period (PRP) paradigm. Thus, we associated either a self-relevant stimulus *or* a negative valent stimulus with a geometric shape as well as two neutral stimuli with other shapes following the matching paradigm as in Experiment 2. Subsequently, we conducted the matching task as one of two tasks in the PRP paradigm. Thereby, the results should allow for an assessment of where each factor influences the successive stages of stimulus processing (Sternberg, 1969), in other words whether it influences stimulus processing at an early, pre-central stage or at a later stage (Pashler, 1994). We expect to find a different data pattern for the self-relevant combination than for the negative-associated combination, indicating that both factors influence stimulus processing in different ways (for more detailed hypotheses, see the methods section of Experiment 3).

Note that, in the first two experiments, we added positive stimuli as a further condition to test for the specificity of effects of self-relevant and negative stimuli. Although a recent meta-analysis indicates evidence for attentional biases for positive compared to neutral stimuli (Pool et al., 2016), effects for positive items were modest, especially in the emotional Stroop task (Bertels & Kolinsky, 2015; Kahan & Hely, 2008).

## Experiment 1

In order to compare the automatic attention-allocating effects of self-relevant and negative stimuli, we presented self-relevant pronouns as well as neutral pronouns and negative, positive, and neutral nouns as words in a color-naming task (i.e., the emotional Stroop task). The specific interference effects due to self-relevance or negative valence were compared. Therefore, performance on self-relevant words was compared to the performance on neutral pronouns and the performance on negative words was compared to the performance on neutral nouns.

### Method

#### Participants

Ninety-one students from the University of Trier (64 female) took part in the experiment, receiving course credit. The data of one participant (female) had to be discarded due to a red-green deficiency. The data of a further participant (female) had to be discarded before analysis because of far too many errors (i.e., far-out value according to Tukey, 1977). Thus, the total sample size was *N* = 89. For this sample, the median age was 22 years (range 18–30). All participants had normal or corrected-to-normal vision. For an a priori calculation of the required sample size, note that emotional Stroop effects were modest in previous studies (*d*_*z*_ > 0.32–0.39 in Frings et al., 2010). A sample of *N* = 89 allows testing for effects of *d*_*z*_ = .30 with a power of 1 - β = .80 (α = .05; G*Power 3.1.9, Faul et al., 2007).

#### Design

The experiment comprised a one-factorial, repeated-measures design with the within-participant factor word category (*self-relevant* vs. *negative* vs. *positive* vs. vs. *neutral nouns* vs. *neutral pronouns*).

#### Material and apparatus

The experiment was conducted using standard PCs with TFT monitors that had a display resolution of 1,280 × 1,024 pixels and standard German QWERTZ keyboards, and by using E-Prime 2.0 software. The words in the negative, positive, and neutral conditions were selected and classified according to an a priori-based valence rating (Wentura, 1998), which revealed a mean rating of 0.04 (*SD* = 0.05) in the neutral condition, -2.67 (*SD* = 0.23) in the negative condition, and 2.55 (*SD* = 0.18) in the positive condition on a scale from -3 (negative) to +3 (positive). In detail, the German words Hose [trousers], Herd [stove], Brett [board], and Mast [pole] were used in the neutral-nouns condition; Gift [poison], Mord [murder], Krieg [war], and Tod [death] in the negative condition; Blume [flower], Leben [life], Liebe [love], and Glück [fortune] in the positive condition. In the self-relevant condition, the German words Ich [I], Mein [my], Mich [me], and Mir [mine] were used. A neutral-pronoun condition was added as a control condition to account for the fact that negative and positive words were nouns whereas self-relevant words were pronouns. Thus, as a control condition for the self-relevant condition, the German words Er [he], Sie [she], Es [it], and Ihr [you (plural)] were added. Due to the small number of appropriate self-relevant labels, we chose four neutral pronouns to equalize the number of words per category.

The words were written in Courier New and were presented in the middle of the screen on a black background. Furthermore, they were presented in yellow (RGB values: 250, 255, 45), green (130, 230, 70), blue (10, 50, 250), and purple (170, 50, 250). For the duration of the experiment, the viewing distance of about 60 cm was controlled with chin rests and resulted in a visual angle of about 0.95° for the words.

#### Procedure

Participants were tested individually in sound-proofed chambers. They were instructed (on the screen and summarized by the experimenter in the beginning of the experiment) to classify the color of each presented word via keystroke: yellow with the V-key (index finger of the left hand), green with the N-key (index finger of the right hand), blue with the F-key (middle finger of the left hand), and purple with the J-key (middle finger of the right hand).

The experiment started with a practice phase, in which numerals (the German words Eins [one], Zwei [two], Drei [three], Vier [four], Fünf [five], Sechs [six], Sieben [seven], and Acht [eight]) were presented in the above-mentioned colors and participants were instructed to react as fast and accurately as possible with the given keys. Each numeral was presented once in each color, resulting in 32 practice trials. In this phase, feedback was given if the participant responded correctly. After the practice phase, the experimental phase started. Here, the words in the five word-category conditions were presented in the mentioned colors. The word categories (neutral nouns, negative nouns, positive nouns, self-relevant pronouns, neutral pronouns) were presented blockwise in order to control for cross-trial effects (for the impact of fast and slow effects in the emotional Stroop task, see Frings et al., 2010; McKenna & Sharma, 2004,); there were 20-s breaks between the blocks. In each word-category block, each of the four words was presented once in each of the four colors, resulting in 16 trials. The five word-category blocks were presented in random order and three times each, resulting in 240 trials in sum in the experimental phase. Each trial, in the practice and in the learning phase, started with a fixation cross for 500 ms after which the word was presented until the participant responded, followed by an inter-stimulus interval of 30 ms. After a 20-s break between the word-category blocks, a fixation cross was presented for 1,500 ms before the next block started. Within the blocks, trials were presented in random order.

### Results

Only correct responses with reaction times (RTs) above 200 ms and below three interquartile ranges above the third quartile of the individual RT distribution (Tukey, 1977) were used for the RT analysis. Averaged across participants, 94.0% of the trials were selected for RT analysis, 5.2% of the trials were excluded because of erroneous responses, and 0.8% were excluded due to the RT outlier criteria. Note that neither the mean RTs nor the mean error rates in the two neutral conditions (i.e., the neutral-noun and the neutral-pronoun condition) differed significantly, both *t*s < 1.08, both *p*s > .283. Hence, the two conditions were merged to a single neutral control condition. Mean RTs and error rates in the resulting conditions are shown in Table 1.

**Table 1:** Mean reaction times (RTs; in milliseconds) and error rates (in %) in the emotional Stroop task as a function of word category (*self-relevant* vs. *negative* vs. *positive* vs. *neutral_nouns* vs. *neutral_pronouns*). Standard deviations are given in parentheses

		RTs	Error rates
Word category	Self-relevant	592 (73)	1.4 (1.4)
	Negative	602 (81)	1.3 (1.2)
	Positive	593 (75)	1.2 (1.2)
	Neutral_nouns	594 (79)	1.4 (1.4)
	Neutral_pronouns	592 (75)	1.2 (1.2)

#### Reaction times

As can be seen from Table 1, negative words are associated with larger mean RTs compared to all other conditions. To address the hypotheses, interference effects were assessed by comparing mean RTs in the neutral condition and mean RTs in the negative, positive, and self-relevant condition, respectively. These differences were represented by a priori simple contrasts (with neutral trials as the reference) in a one-factorial (word category: *neutral* vs. *negative* vs. *positive* vs. *self-relevant*) repeated-measures MANOVA with mean RTs as the dependent variable (for the use of MANOVA analyzing repeated-measures designs, see O’Brien & Kaiser, 1985). In this analysis, the overall effect of word condition was associated with *F*(3, 86) = 2.25, *p* = .088, η_p_^2^ = .073. The interference effect in the negative condition was significant, *F*(1, 88) = 5.14, *p* = .026, η_p_^2^ = .055, representing a typical emotional Stroop effect. Remarkably, there was no such interference effect in the self-relevant condition, *F* < 1 (Fig. 1).

Additionally, mean RTs in the self-relevant and in the negative condition were significantly different, *t*(88) = 2.34, *p* = .022, *d*_*z*_ = 0.25, indicating a significantly larger interference effect in the negative condition than in the self-relevant condition. For the sake of completeness, note that the simple contrast positive versus neutral was non-significant, *F* < 1.

#### Error rates

A one-factorial (word category: *self-relevant* vs. *negative* vs. *positive* vs. *neutral*) repeated-measures MANOVA with error rates as the dependent variable revealed no significant effects (all *p*s > .140), indicating that there was no effect of word condition on error rates.

### Discussion

The combination of self-relevant, negative, positive, and neutral stimuli in the emotional Stroop task revealed an effect of negative stimuli on RTs, indicating the expected emotional Stroop effect. Above that, there was no indication for such an effect for self-relevant stimuli and there was a significant difference between the automatic attention allocation due to negative stimuli compared to the automatic attention allocation due to self-relevant stimuli. Consequently, the data pattern observed in the emotional Stroop paradigm revealed a first dissociation between effects of self-relevance and effects of negative valence.

However, as with most emotional Stroop tasks, our task had some caveats (see, e.g., Larsen et al., 2006) since one cannot use the same word material in the different conditions. Put even more strongly, emotional Stroop tasks are inherently quasi-experimental (as you will typically observe differences in word frequency, word length, orthographic-neighborhood density, and so on). With regard to our experiment, the self-relevant stimuli (and associated control stimuli) consisted of pronouns; the affective stimuli comprised nouns. In addition, the word length between these stimuli and in turn the average word length per condition differed as well as the word frequency. Thus, in principle, differences in linguistic characteristics of the material may have contributed to the observed data pattern even if the non-significant difference between responses in the two neutral conditions (both *t*s < 1.08, both *p*s > .283) contradicts an effect of these factors in our data given the differences of lexical characteristics between these two conditions.

## Experiment 2

In order to assess whether the influence of self-relevance can be distinguished from influences of negative stimuli, we compared effects of self-relevant and negative associations in a paradigm in which prioritization by associative learning can be measured. Thus, we adapted the matching paradigm and assigned four neutral geometric forms to a self-relevant, a highly negative, a highly positive, or a neutral stimulus. The prioritization effects of interest were measured as the difference between performance in the self-relevant or the negative condition compared to performance in a neutral control condition.

### Method

#### Participants

Forty students from the University of Trier (34 female) took part in the experiment receiving course credit. Median age was 21 years (range 18–39) and they all had normal or corrected-to-normal vision. As the typically measured prioritization effect in this paradigm, the SPE with visual stimuli, was rather large in previous studies (*d*_*z*_ > 0.65 in Schäfer et al., 2015; Sui et al., 2012), with a sample of *N* = 40 we tested for effects of *d*_*z*_ = .60 with a power of 1 - β = .96 (two-tailed, α = .05; G*Power 3.1.9, Faul et al., 2007).

#### Design

Experiment 2 comprised a 2 (matching condition: *matching* vs. *non-matching*) × 4 (shape: *self-associated* vs. *negative-associated* vs. *positive-associated* vs. *neutral-associated*) within-participants design. The assignment of the shapes to the labels was balanced across participants following a Latin-square design.

#### Material and apparatus

The situation in the laboratory was the same as in Experiment 1. The geometric shapes were a square, a circle, a triangle, and a rectangle, and were associated either with the German word Ich [I] as the self-relevant label or with one of ten words with distinct valence in the negative, positive, and neutral condition. Balanced across participants, a negative, a positive, and a neutral label were chosen out of the following words: Folter [torture], Mord [murder], Krieg [war], Henker [executioner], Unfall [accident], Gewalt [violence], Gift [poison], Pest [plague], Sadist [sadist], and Horror [horror] in the negative condition; Musik [music], Blume [flower], Leben [life], Liebe [love], Meer [sea], Urlaub [holiday], Natur [nature], Sommer [summer], Freund [friend], and Lachen [laugh] in the positive condition; and Wand [wall], Klinke [handle], Balken [beam], Hose [trouser], Brett [board], Tisch [table], Herd [stove], Teller [plate], Lampe [lamp], and Boden [floor] in the neutral condition. As in Experiment 1, the labels in the negative, positive, and neutral conditions were selected and classified according to an a priori valence rating (Wentura, 1998), which revealed a mean rating of -2.56 (SD = 0.22) for the negative words, 2.60 (SD = 0.14) for the positive words, and 0.05 (SD = 0.11) for the neutral words. Additionally, a mean valence rating subsequent to the experiment (-3 = negative, +3 = positive) was -2.15 (SD = 1.3) for the negative words, 2.08 (SD = 1.3) for the positive words, and -0.03 (SD = 0.8) for the neutral words. All stimuli were presented in white on black background. The labels were presented in Courier New, with a viewing distance of about 60 cm resulting in a visual angle of about 0.57°. The geometric shapes, the labels, and a fixation cross were presented from the centre of the computer screen, subtending 4.3° × 4.3° visual angle for the geometric shapes (except for the rectangle which was 4.3° visual angle high and 8.6° wide).

#### Procedure

As in Experiment 1, participants were tested individually in sound-proofed chambers and task instructions were given on the screen and summarized by the experimenter. The experiment started with a learning phase, in which the to-be-learned assignments were shown on the display for 60 s in written form. For a particular participant this might read: “I am a triangle. Poison is a circle. Music is a square. And trouser is a rectangle.” Participants were instructed to place the index finger of the left hand on the S-key (non-matching response) and the index finger of the right hand on the L-key (matching response).

After the learning phase, the matching task began. Here, each trial started with a 500-ms presentation of a black screen, followed by a fixation cross for 500 ms. Then a pairing of one of the labels and one of the geometric shapes was presented for 100 ms, followed by a black screen until the participant responded or 1,500 ms had elapsed. Participants’ task was to judge whether the displayed label-shape pairing corresponded to one of the initially learned assignments or not. One experimental session consisted of a short practice block with 24 trials (in which feedback was given on the screen) and an experimental block with 240 trials (without feedback). In the experimental phase, each geometric shape was presented in 60 trials. Half of the trials depicted matching and half of them non-matching assignments. The same proportions were realized in the practice phase. Trials were presented in random order.

### Results

Only correct responses with RTs above 200 ms and below three interquartile ranges above the third quartile of the individual RT distribution (Tukey, 1977) were used for the RT analysis. Averaged across participants, 83.4% of the trials were selected for RT analysis, 16.0% of the trials were excluded because of erroneous responses, 0.6% due to the RT outlier criteria. Mean RTs and error rates are shown in Table 2.

**Table 2:** Mean reaction times (in milliseconds) and error rates (in %) in the matching paradigm as a function of matching condition (*matching* vs. *non-matching*) and shape association (*self* vs. *positive* vs. *negative* vs. *neutral)*. Standard deviations are given in parentheses

		Matching condition			
		Matching		Non-matching	
		RTs	Error rates	RTs	Error rates
Shape association	Self	605	1.6 (2.2)	726	2.0 (1.8)
	Negative	666	2.0 (2.1)	725	2.2 (1.7)
	Positive	672	1.9 (1.8)	734	2.4 (1.9)
	Neutral	675	2.2 (1.9)	732	1.8 (1.5)

#### Reaction times

We conducted the overall 2 (matching condition: *matching* vs. *non-matching*) × 4 (shape association: *self* vs. *negative vs. positive* vs. *neutral*) repeated-measures MANOVA with mean RTs as the dependent variable. The main effect of matching condition was associated with *F*(1,39) = 114.32, *p* < .001, η_p_^2^ = .75, indicating significantly faster responses in matching trials. The main effect for shape association was associated with *F*(3,37) = 4.91, *p* = .006, η_p_^2^ = .29, indicating a difference in the RTs due to the shape association. The interaction was also significant, *F*(3,37) = 7.16, *p* < .001, η_p_^2^ = .37, showing that the effect of the shape association was different in matching than in non-matching trials.

Prioritization effects are usually analyzed in matching trials, because matching and non-matching trials involve different processes and prioritization has most reliably been demonstrated in matching trials (see, e.g., Humphreys & Sui, 2016). Following this, the hypothesized prioritization effects were assessed by the difference between mean RTs in the neutral-associated trials and mean RTs in the self- *or* negative-associated trials in the matching condition. These two comparisons corresponded to simple contrasts in a one-factorial, repeated-measures MANOVA in the matching condition with the factor shape association (*self* vs. *negative* vs. *positive* vs. *neutral*) and with mean RTs as the dependent variable. The prioritization effect of the self-associated condition was significant, *F*(1, 39) = 20.30, *p* < .001, η_p_^2^ = .34, whereas the prioritization effect in the negative-associated condition was not, *F* < 1. This result suggests that only self-relevant associations were prioritized, but negative associations were not (Fig. 1). Finally, a comparison of the two prioritization effects revealed a significant difference, *t*(39) = -3.91, *p* < .001, *d*_*z*_ = .62, showing that the prioritization of self-relevant associations was significantly stronger than the prioritization of negative associations. The main effect of shape association was associated with *F*(3, 37) = 7.87, *p* < .001, η_p_^2^ = .39. For the sake of completeness, note that the simple contrast for positive items was not significant either, *F* < 1, contradicting a prioritization effect of positive associations.

#### Sensitivity measures

Accuracy rates were analyzed computing signal detection-sensitivity indices (*d’*) for each shape condition. Correct responses in matching trials were considered hits, whereas erroneous responses in non-matching trials were considered false alarms. We followed the log-linear approach to account for cases with 100% hits or 0% false alarms (Hautus, 1995; Stanislaw & Todorov, 1999) when computing the *d’* indices. A one-factorial repeated-measures MANOVA with *d’* as the dependent variable and the within-participant factor shape (*self-associated* vs. *negative-associated* vs. *positive-associated* vs. *neutral-associated*) revealed a significant prioritization effect (indicated by the simple contrast with the neutral control condition) only for the self-associated condition, *F*(1, 39) = 6.39, *p* = .016, η_p_^2^ = .14, and not for the negative-associated condition, *F* < 1 (Fig. 1). A comparison of these two prioritization effects revealed a significant difference, *t*(39) = 2.34, *p* = .024, *d*_*z*_ = .37, showing that the prioritization of self-relevant associations was significantly stronger than the prioritization of negative associations. The main effect was associated with *F*(3, 37) = 3.07, *p* = .040, η_p_^2^ = .20, indicating that sensitivity varied according to the shape. Again note that the simple contrast for positive associations was not significant either, *F* < 1, contradicting a prioritization effect of positive associations. Taken together, the analysis of the sensitivity measures confirmed the results of the RT analysis.

### Discussion

In Experiment 2, the analysis of RTs and sensitivity revealed a prioritization effect of self-relevant associations (as in previous studies) that was significantly larger than the (non-significant) prioritization effect of negative associations. Thus, the effect of self-relevance on association learning was different from the effect of negative valence.

Generally speaking, one might argue that again lexical differences in the word material constitute a problem. Yet, in Experiment 2, these differences are less problematic than in Experiment 1. Regarding this, there is evidence that word length and word frequency do not influence performance in the matching task as an SPE has been shown even with the label “yourself” instead of “you,” which was less frequent and longer than the used control stimuli (Exp. 3 in Sui et al., 2012). Yet, there is evidence for an effect of word concreteness (Wade & Vickery, 2017) as well as for of grammatical distinctiveness (Schäfer, Wentura, & Frings, 2017) on prioritization in the matching task. However, there is no indication how the material characteristics of the used material can explain the differences between the self and negative conditions altogether. Nevertheless, we conceptually replicated the difference in processing of negative and self-relevant stimuli in Experiment 3, with another task that is even less susceptible to lexical differences.

## Experiment 3

So far, two experiments indicate different effects of self-relevance and negative valence. We decided to conduct a third experiment, which is as comparable as possible with regard to the conditions under which the two stimulus types influence information processing. Again, we used the matching paradigm as in Experiment 2. However, in a between-participants design, we now contrasted one self-relevant *or* one negative valent stimulus to two neutral stimuli each, so that the stimulus type of interest (either self-relevant or negative) is always contrasted to two neutral control stimuli. Thus, while Experiment 2 clearly indicated that in direct competition the self-relevant stimuli are prioritized and the negative are not, Experiment 3 puts the question of whether prioritization is unique to self-relevant stimuli to a further test. Most important, we embedded the matching task into the PRP paradigm to obtain a specific signature of the effects of self-relevance and negative valence. In this regard, a specific signature for the self-prioritization effect has recently been found (Janczyk et al., 2019), as we explain below.

In the PRP paradigm, two tasks are performed partially overlapping on each trial. The degree of their overlap is manipulated by the stimulus-onset asynchrony (SOA), that is, the time between the onsets of the two stimuli demanding two different responses. Typically, RTs in Task 2 (RT2) depend on the SOA in that participants are slower with short SOAs (i.e., when both tasks are started shortly one after the other) – the PRP effect (Telford, 1931). One influential model to account for the PRP effect is the central-bottleneck model (Pashler, 1994). Based on Sternberg’s notion of successive stages of stimulus processing (Sternberg, 1969), the central-bottleneck model assumes that whereas pre- and post-central stages of stimulus processing can run in parallel with all other processes, only one *central* process can run at any given time. Thus, at the central stage of stimulus processing, a bottleneck occurs (Pashler, 1994). Consequently, at short SOAs, that is when processing of both stimuli happens rather in parallel, a cognitive slack arises, indicated by longer RTs. In contrast to that, at long SOAs, no cognitive slack occurs and the processing of Task 2 is not interrupted.

A typically tackled question is whether a particular experimental effect is influenced by the cognitive slack or not. This influence is indicated by a moderation of the effect of interest by the SOA manipulation – as a cognitive slack only occurs at short SOAs. Specific conclusions can be drawn based on the dependence or independence of the particular effect on the cognitive slack. More specifically, if an effect of interest is *not* affected by the SOA manipulation, then this independence of the cognitive slack is interpreted in terms of the effect influencing stimulus processing after the cognitive slack (i.e., at a central or post-central stage). Contrary to that, if an effect of interest is affected by the SOA manipulation, it can be argued that this effect influences stimulus processing before the cognitive slack (i.e., at the pre-central, perceptual stage; see, e.g., Fischer & Schubert, 2008; Janczyk et al., 2014; Pashler, 1994).

Given this logic, Janczyk et al. (2019) used the matching task as Task 2 in the PRP paradigm. (Task 1 was a simple tone discrimination.) In four experiments, the results consistently demonstrated a PRP effect (i.e., responses in the matching task were slower in the short compared to the long SOA). However, with the same consistency, they found that the SPE occurs at short as well as at long SOAs. This finding suggests that self-relevance affects stimulus processing at a late stage, definitely not at an early perceptual stage.

Thus, in Experiment 3, in one sample, we tested for the effect of a self-relevant stimulus (in comparison to a neutral condition) and tested whether a potential effect is moderated by the SOA manipulation. In a second sample, everything was the same except that we replaced the self-related stimulus with a negative stimulus to test for a potential effect of negative valence as well as whether this effect is moderated by SOA.

We hypothesized replicating the finding by Janczyk et al. (2019), hence to find no significant interaction of the effect of self-relevance with SOA, which highlights self-relevance as a late process. Furthermore, subsequent to Experiment 2, Experiment 3 is a second test of a negativity prioritization effect. Thus, we hypothesized that negative valence affects stimulus processing at a pre-central stage and that its effect should thereby be influenced by the cognitive slack. Thus, we hypothesized no effect of SOA on the processing of self-relevance, but a significant effect of SOA on the processing of negative valence.

### Method

#### Participants

Thirty-eight students from the University of Trier (27 female) took part in the experiment receiving course credit. Median age was 21 years (range 18–27). They had normal or corrected-to-normal vision and reported no hearing difficulties. Participants were randomly assigned to one of the two relevance conditions (which resulted in *n*_*1*_ = 20 for the self-related condition and *n*_*2*_ = 18 for the negative condition).

The SPEs in Experiments 1a, 1b, and 2 by Janczyk et al. (2019; which are directly comparable to our Exp. 3) were in the range η_p_^2^ = .50–.58. Assuming η_p_^2^ = .50, a sample of *n* = 8 is needed for power 1-β = .80 (α = .05). With *n*_*2*_ = 18 in the negative-valence condition, we had a power of 1-β = .80 (α = .05) to detect an effect of the size η_p_^2^ = .24 (be it for the negativity-prioritization effect or for the interaction of this effect with SOA).

#### Design

Experiment 3 comprised a 2 (relevance condition: *self-relevant* vs. *negative valence*) × 3 (shape: *relevant-associated* vs. *neutral1-associated* vs. *neutral2-associated*) × 2 (matching condition: *matching* vs. *non-matching*) × 2 (SOA: *short* vs. *long*) mixed design. The assignment of the shapes to the labels was balanced across participants following a Latin-square design.

#### Material and apparatus

As a whole, methodological details were chosen to replicate the experiments by Janczyk et al. (2019). Hence, experimental procedures were controlled by a standard PC and the first stimulus was either a 300- or a 900-Hz tone (50 ms) presented via headphones. The geometric shapes were a square, a circle, and a triangle, and all stimuli were presented in white against a black background. Adjusted to the current research question, the associated words were Stuhl [chair] and Baum [tree] as the two neutral labels.^1^ The relevant label in the self-relevant condition was the word Ich [I]; the relevant label in the negative-valence condition was one of the three negatively connoted words Folter [torture], Krieg [war], or Gewalt [violence] (balanced across participants in this condition). The labels were presented in Courier New font, with a viewing distance of about 60 cm, resulting in a visual angle of about 0.5°. The geometric shapes, the labels, and a fixation cross were presented from the centre of the computer screen, subtending 2.9° × 2.9° visual angle for the geometric shapes.

#### Procedure

As in Experiment 2, the experiment started with a learning phase, in which the to-be-learned assignments were shown for 60 s in written form. For a particular participant this might read: “I am a triangle. The chair is a circle. The tree is a square.” (“Torture is a triangle. The chair is a circle. The tree is a square.” in the negative-valence condition). Participants were instructed to place the middle finger of the left hand on the S-key and the index finger of this hand on the D-key (response keys for Task 1) and the middle and index finger of the right hand on the K- and L-keys, respectively (response keys for Task 2). After this learning phase, the PRP paradigm started. Here, the first task was an auditory discrimination task and the second task was the matching task. Following a fixation cross (500 ms), a tone was played for 50 ms. Either 100 ms or 1,000 ms after the onset of the tone, a label-shape combination appeared for 300 ms. After that, a blank screen appeared for 2,200 ms or until the participant finished both responses. Error feedback was provided for 1,000 ms if the participant entered the second response first. A response on a high tone was given with the S-key, on a low tone with the D-key, and a response on a matching combination was given with the K-key and non-matching with the L-key. One block consisted of 48 trials, resulting from the combination of the 2 (S1: 300 vs. 900 Hz) × 2 (SOA: 100 vs. 1,000 ms) × 3 (shape: triangle, square, circle) × 3 (label: relevant vs. neutral1 vs. neutral2) conditions, whereby matching label-shape combinations were presented twice as often as each possible non-matching combination in order to have the same proportion for matching and non-matching trials (Sui et al., 2012). Ten repetitions resulted in 480 trials in sum, and after 160 and 320 trials, a break slide appeared, which the participants could end as soon as they wanted to go on. Ten randomly chosen trials were presented as practice trials before the experimental phase started.

### Results

Erroneous trials (either wrong response, no response given within 2,500 ms after the onset of the second stimulus, or the second response given first) were excluded from the RT analyses. Further, responses below 200 ms and above 3 interquartile ranges above the third quartile of the overall RT distribution (Tukey, 1977) were excluded. As the effect of the cognitive slack can be seen in a possible interruption of the second task, analysis in the PRP paradigm focuses on the performance in this task (for the sake of completeness, we briefly report the data pattern in the first task).

#### Matching task (Task 2)

For an analysis of the performance in the second task, averaged across participants, 73.6% of the trials were selected for RT analysis; 26.4% of the trials were excluded because of erroneous responses and no trials were excluded due to the RT-outlier criteria. Mean RTs and error rates are shown in Table 3.

**Table 3:** Mean RTs (in milliseconds) and error rates (in %) in the second task of the psychological-refractory-period (PRP)-paradigm as a function of matching condition (*matching* vs. *non-matching*), relevance condition (*self-relevant* vs. *negative valence*), shape (*relevant-associated* vs. *neutral1-associated* vs. *neutral2-associated*), and stimulus-onset asynchrony (SOA; *100 ms* vs. *1,000 ms*). Standard deviations are given in parentheses

			SOA			
			100 ms		1,000 ms	
Matching condition	Relevance condition	Shape	RTs	ERs	RTs	ERs
matching	self-relevant	relevant	1342 (233)	36.5 (23.8)	860 (189)	18.5 (19.4)
		neutral1	1438 (237)	36.5 (23.8)	980 (207)	24.3 (18.7)
		neutral2	1427 (251)	43.4 (16.3)	999 (260)	24.6 (19.0)
	negative valence	relevant	1259 (243)	31.4 (22.1)	839 (184)	19.0 (25.5)
		neutral1	1300 (252)	33.3 (23.3)	852 (222)	20.5 (27.7)
		neutral2	1295 (236)	33.8 (22.1)	795 (146)	20.4 (26.9)
non-matching	self-relevant	relevant	1428 (254)	32.0 (17,9)	989 (218)	17.5 (20.0)
		neutral1	1507 (250)	31.5 (15.5)	1065 (223)	19.5 (17.7)
		neutral2	1446 (232)	35.1 (18.6)	1046 (213)	16.6 (18.6)
	negative valence	relevant	1328 (229)	27.4 (16.4)	905 (202)	16.9 (20.2)
		neutral1	1364 (243)	26.1 (20.5)	918 (195)	16.9 (21.6)
		neutral2	1344 (246)	26.5 (20.5)	891 (184)	17.5 (20.1)

**Fig. 2:**
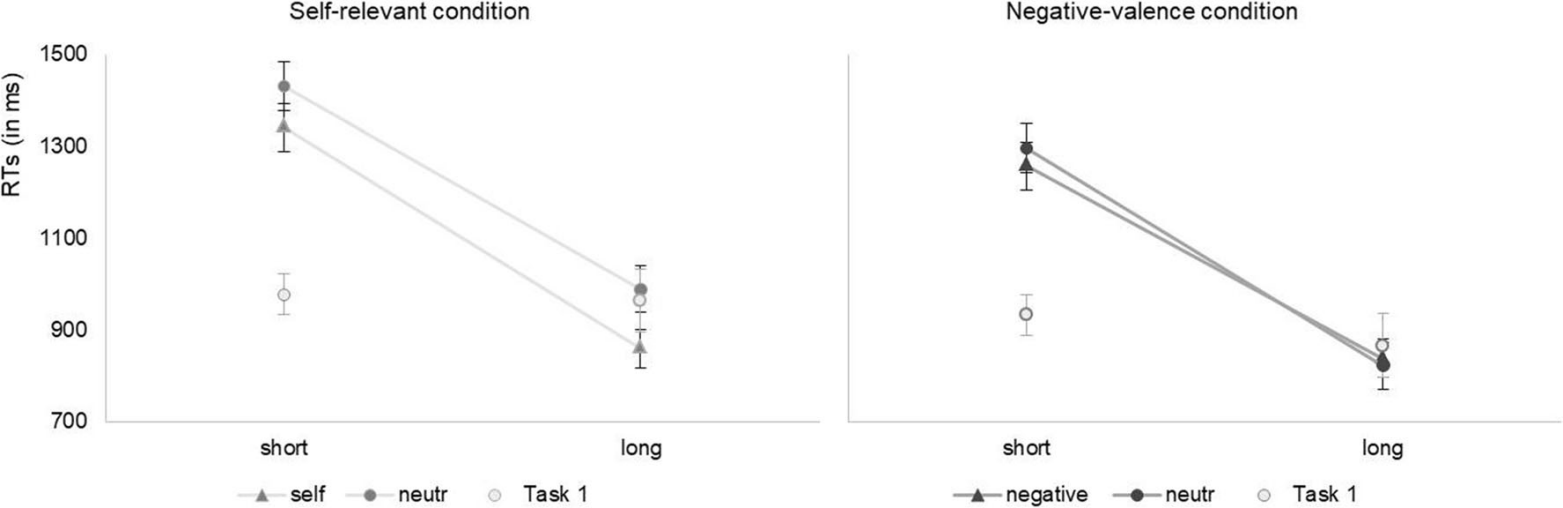
Mean RTs in the relevant-associated condition in the self-relevant and negative-valence conditions in comparison with the particular neutral-associated condition (circles in the particular color), as well as mean RTs in the first task in each SOA condition for inspection. Error bars indicate standard errors of the means

Inferential statistics of the overall 2 (relevance condition: *self-relevant* vs. *negative valence*) × 3 (shape: *relevant-associated* vs. *neutral1-associated* vs. *neutral2-associated*) × 2 (matching condition: *matching* vs. *non-matching*) × 2 (SOA: *short* vs. *long*) MANOVA for repeated measures with relevance as a between-participants factor and mean RTs as the dependent variable are reported in the Appendix (Appendix 1). As explained in Experiment 2, prioritization effects in the matching paradigm are analyzed in matching trials. Further, as in Experiment 2, RTs in the two neutral-associated conditions were averaged because the two neutral labels represented the same condition. Thus, to test our hypotheses, we conducted a 2 (relevance condition: *self-relevant* vs. *negative valence*) × 2 (shape: *relevant-associated* vs. *neutral-associated*) × 2 (SOA: *short* vs. *long*) mixed-design MANOVA. The main effect of shape was significant, *F*(1, 36) = 13.73, *p* = .001, η_p_^2^ = .28, indicating overall faster responses in the relevant-associated trials compared to the neutral-associated trials, and thereby demonstrating an effect of the newly built associations – that is, faster responses in the relevant-associated condition in comparison to the neutral-associated condition. Further, responses were significantly faster with long than with short SOAs, *F*(1, 36) = 352.26, *p* < .001, η_p_^2^ = .90, demonstrating that participants had to solve the cognitive slack at short SOAs (thus, a large PRP effect). There was no significant main effect of the between-subject factor relevance condition, *F*(1, 36) = 2.53, *p* = .121, η_p_^2^ = .07. There was a significant interaction of association and relevance condition, *F*(1, 36) = 8.93, *p* = .005, η_p_^2^ = .20, indicating that the effect of the association depended on the relevance condition. Remarkably, there was a significant three-way interaction, *F*(1, 36) = 5.46, *p* = .025, η_p_^2^ = .13, which indicates that the benefit due to self-relevance (i.e., faster responses in the relevant condition than in the neutral condition) and the benefit due to negative valence are differently influenced by SOA. See Fig. 2 for the data pattern in both relevance conditions. No other interaction was significant, both *F*s < 1.

In order to investigate whether each of the two effects is influenced by the cognitive slack or not, in other words, whether each of the two effects influences stimulus processing *before* (i.e., at a precentral stage) or *after* the cognitive slack (i.e., at a central or post-central stage), we tested for the specific interactions with SOA. Hence, separately for the two relevance conditions, we calculated two 2 (shape: *relevant-associated* vs. *neutral-associated*) × 2 (SOA: *short* vs. *long*) MANOVAs. For the self-relevant condition, this analysis revealed two significant main effects, *F*(1, 19) = 28.6, *p* < .001, η_p_^2^ = .60 for the association and *F*(1, 19) = 193.5, *p* < .001, η_p_^2^ = .91 for SOA, revealing an effect of the association with self-relevance as well as a PRP effect. Most importantly, there was no significant interaction, *F*(1, 19) = 1.64, *p* = .216, η_p_^2^ = .08, revealing an independence of the effect of self-relevance from the cognitive slack and thereby replicating previous findings (Janczyk et al., 2019). In contrast to this, for the negative-valence condition, the same analysis revealed a significant main effect of SOA, *F*(1, 17) = 160.6, *p* < .001, η_p_^2^ = .90, revealing a PRP effect, but no overall effect of the association with negative valence (as indicated by the non-significant main effect of association, F < 1). Remarkably, there was a significant interaction of the association and SOA, *F*(1, 17) = 4.74, *p* = .044, η_p_^2^ = .22. In detail, with long SOAs, there was no negative-prioritization effect, *M* = -16 ms (*SD* = 93 ms), *t*(17) = -0.71, *p* = .488, *d*_*z*_ = 0.17. With short SOAs, numerically a negative-prioritization effect was present, which, however, was not significant, *M* = 39 ms (*SD* = 145 ms), *t*(17) = 1.14, *p* = .271, *d*_*z*_ = 0.27. For the sake of full transparency, it might be of interest that this difference variable was burdened by an outlier. To adequately account for this, we tested the negative-prioritization effect in a t-test for trimmed means (see, e.g., Wilcox, 1997) with a trimming of γ = .20; it yielded *t*(9) = 2.26, *p* = .050.

Comparable analyses were conducted with error rates and revealed no contradicting effects. The 2 (relevance condition: *self-relevant* vs. *negative valence*) × 2 (shape: *relevant-associated* vs. *neutral-associated*) × 2 (SOA: *short* vs. *long*) mixed-design MANOVA revealed a significant main effect of the shape, *F*(1, 36) = 7.26, *p* = .011, η_p_^2^ = .17, demonstrating an effect of the newly built associations by more accurate responses in the relevant-associated condition in comparison with the neutral-associated condition (see Table 3). Moreover, the main effect of SOA was significant, *F*(1, 36) = 30.75, *p* < .001, η_p_^2^ = .46, indicating the PRP effect. No other effects were significant, all *F*s < 2.14, all *p*s > .152.

#### Tone task (Task 1)

For the performance in the first task (tone-discrimination task), we checked for the overall performance by assessing the distribution of mean errors for all participants. Based on the same exclusion criteria as in Task 2, 92.9% of the trials were selected for RT analysis; 7.1% of the trials were excluded because of erroneous responses and no trials were excluded due to the RT-outlier criteria. Means for RTs and errors are given in Table 4 (Appendix 2; for mean RTs as a function of SOA condition, see Table 4). Inferential statistics for the overall MANOVA are also reported in Appendix 2. Of interest for the discussion is the conclusion that a precondition for applying the PRP logic – that is, no moderation of Task 1 performance by SOA due to the instructed prioritization of Task 1 – holds in the sample presented with the self-relevant matching task whereas it seems to be violated in the sample presented with the negative-valence matching task: In the latter sample, Task 1 responses are slower with short SOAs than with long SOAs.

### Discussion

First, Experiment 3 corroborates the results of Experiment 2 in a between-participants design: Again, a strong self-prioritization effect was found, but no comparable negative-prioritization effect. Thus, even if the negative stimulus does not directly compete for prioritization with the self-relevant stimulus, no prioritization was found. This is most evident in the long SOA condition, which is roughly comparable to the standard matching task. Second, Experiment 3 clearly replicated a recent study by Janczyk et al. (2019). In the self-relevant condition, no interaction of the effect of self-relevance and SOA was found. This non-significant interaction shows that self-relevance and SOA influence stimulus processing additively, and this indicates a late process of self-relevance (Janczyk et al., 2019). Third, in contrast to this finding, in the negative-valence condition, a significant interaction of the effect of negative valence with SOA was found. Thus, before elaborating on this interaction, we can state that, in a completely comparable setting, self-relevance influences stimulus processing in a different manner than negative valence does.

Remarkably, however, the interaction found for negative stimuli does not fit the usual central-bottleneck logic of the PRP paradigm. A usual interpretation of a moderation of an experimental effect by SOA is that this particular effect influences stimulus processing at an early stage and therefore falls into the cognitive slack with short SOA (locus-of-slack logic). Consequently, the effect should be absent with a short SOA, but present with a long SOA. This was not the case in our experiment: We observed larger negative-neutral differences with short than with long SOAs. Thus, the data pattern we found does not correspond to the central-bottleneck model, but rather indicates capacity sharing – a further explanation for the effects in the PRP paradigm (Pashler, 1994; see also Schneider et al., 2020, for a smilar logic). According to capacity-sharing models, the performance of more than one task at any given moment results in less capacity for each individual task so that performance is impaired.

In this regard, it is noteworthy that, in the negative-valence condition, responses in Task 1 were (in general, i.e., irrespective of the concrete Task 2 stimuli) slowed down in the short SOA condition compared to the long SOA condition. This moderation of Task 1 performance by SOA suggested that participants refrained from the instructed prioritization of Task 1. This might indicate that an expectation of the processing of negative valence in Task 2 reduced the prioritization of Task 1. In this specific context, we found a hint of a negative-prioritization effect. In other words, negativity prioritization is only found if processing of the negative stimulus is in competition to a different task. This interpretation fits with other findings for negative stimuli. Typically, negative stimuli are task-irrelevant and detract from a main task – for example, as in the emotional Stroop task of our Experiment 1. This detraction can result in two effects, in performance decrements for the main task (i.e., slowed color naming in the emotional Stroop task) and/or in performance facilitation for the negative stimulus itself (e.g., the negative word in the emotional Stroop task might be better encoded than neutral words). In Experiment 3, we see the latter effect in the form of a (weak) negative-prioritization effect. Admittedly, a stimulus-specific performance decrement in Task 1 was not found. However, the simple tone-discrimination task might be not sensitive enough to highlight this effect.

Nevertheless, the results of Experiment 3 again confirm our hypothesis that self-relevance serves as associative glue (e.g., Sui 2016) and that therefore a different pattern of results should be observed for negative and for self-relevant stimuli.

## General discussion

The aim of our study was to compare the processing of self-relevant stimuli and negative stimuli and in particular to further corroborate the assumption of self-relevance as an associative glue, facilitating the formation of associations between stimuli, beyond any attention-grabbing effects self-relevance may have. In that regard, we compared the effects of self-relevant stimuli with effects of negative stimuli in one paradigm in which automatic attentional capture is assumed to be measured (Exp. 1) and in another paradigm in which associative strength is measured (Exp. 2 and Exp. 3). Additionally, in Experiment 3, the effects of self-relevance and negative valence were tested for the particular way they influence stimulus processing.

We found an interference effect due to negative valence in the emotional Stroop paradigm and an effect of self-relevance in the matching paradigm, but not vice versa. The effect of self-relevance in the matching paradigm was replicated in Experiment 3, indicating being post-perceptual in nature through the missing SOA moderation in the PRP paradigm, and again dissociated by the effect of negative valence. In detail, when self-relevant and negative stimuli were presented as task-irrelevant features (as is the case in the emotional Stroop paradigm), negative stimuli caused interference. This interference is assumed to emerge due to the fact that negative valence automatically attracts attention and therefore impedes the relevant response. In contrast, self-relevant stimuli did not cause any interference in the emotional Stroop task. In addition, in Experiment 2, we contrasted self-relevant and negative associations and found that only self-relevant stimuli caused an associative advantage. This double dissociation was further supported by different influences of resource limitation on the effect of self-relevance and negative valence: self-relevance was not influenced at all, indicating that it influences stimulus processing at a later stage, whereas negative valence was influenced significantly, indicating that it influences stimulus processing at an early stage.

As an aside, we did not find an effect of positive valence in the first two experiments (and therefore removed this condition in Exp. 3). As mentioned above, attentional effects for positive stimuli do not necessarily occur in selective-attention paradigms (see, e.g., Bertels & Kolinsky, 2015). In previous studies with the matching paradigm, effects of positive valence due to associations with high reward had been observed. Although similar effects of self-relevance and reward were found, several studies pointed out a difference between these effects (see, e.g., Sui et al., 2015; Sui & Humphreys, 2015a,c). Still, a dominant effect of a self-relevant stimulus beyond a positive as well as beyond a negative stimulus emphasizes different underlying processes and further emphasizes the specific effects of self-relevance.

In that regard, one might also consider the coexistence of effects of self-relevance and valence. Although self-relevant stimuli can potentially be rewarding (and thereby positively valent), self-relevant stimuli might also gain negative valence. In the case of coexistence of self-relevance and negative valence, the influence on attention could be double-edged: allocating attention towards these stimuli as well as strengthening associations between these stimuli and the self. Considering the presumable functionality of the concept of “self-relevance,” the effects of self-relevance and negative valence might also interact rather than influencing stimulus processing additively. Thus, the effects of self-relevant stimuli on cognitive processing might be modulated by their current positive or negative connotation. Still, even such potential results of further research would be in line with our argument here, namely that effects of self-relevance on the one hand and (negative) valence on the other can be separated.

### Theoretical implications

Taken together, the measured effects of self-relevance and negative valence emphasize the special way in which self-relevance guides stimulus processing. While the reported effects confirm negative valence as an attention-allocating factor that represents a general, early selection mechanism (for previous evidence for this assumption, see, e.g., Clarke et al., 2013; Fox et al., 2001; Yiend, 2010), self-relevance is demonstrated to be different from that and, specifically, to boost the learning of arbitrary associations (for previous findings suggesting a learning advantage due to self-relevance, see, e.g., Fuentes et al., 2016; and also Cunningham et al., 2008; Englert & Wentura, 2016; see Sui, 2016 for a theoretical interpretation). The double dissociation of effects of self-relevance and effects of negative valence integrates previous findings as well as theoretical assumptions and, for the first time, allows for the clear interpretation of self-relevance as an associative glue.

Note that this is by no means an indication that self-relevance cannot lead to automatic allocation of attention. Although we did not observe such an effect in the emotional Stroop task used here, it is clear that some previous findings on self-relevance do fit with an interpretation in terms of automatic attention allocation. Additionally, the emotional Stroop task with word material typically leads to weak effects (as compared to pictures; see, e.g., Kunde & Mauer, 2008), so the fact that we did not observe an emotional Stroop effect for self-relevant words does not suggest that self-relevance cannot allocate attention in a bottom-up fashion. More important is the fact that negative stimuli do this to a stronger degree while they at the same time do not lead to an associative learning advantage as self-relevant stimuli do. In addition, self-relevance seems to influence stimulus processing at a *different* – and in particular at a *later* – stage than negative valence. Thus, our point is that there is more to self-relevance than attention allocation.

The idea of an associative effect of self-relevance highlights a potential function of our self-concept: the creation of a network of importance. Once stimuli are perceived as being relevant for our self (obviously also via instruction, as in the association phase in the matching paradigm), their connection to the self-concept is privileged and thereby becomes stronger than self-irrelevant connections. In that regard, self-relevance serves to create a network of those contents and elements, which have a particular relevance, by binding them together. Further support for such a “binding approach” of the self-concept comes from the idea that person-perception is comparable to object perception (Hommel, 2018). Assuming that principles for object perception can be transferred to person-perception, the self would be represented by bindings of particular features, the significance of each feature can be weighted according to the current context, and a direct binding from the object – or in our case the person – goes to action (for an overview of a common object-perception approach, the Theory of Event Coding, see Hommel, 2004). In this context, self-relevance might be seen as a higher-order influence on stimulus processing, and thereby categorized as a controlled influence on stimulus-response-effect episodes in general (see, e.g., Frings et al., 2020).

Our results might also reflect a distinction that may have sometimes been overlooked when attention-grabbing effects of self-relevance or negative valence were analyzed. Besides the organization of stimulus characteristics in automatic and voluntary effects, a further separation is between focused attention on the task versus attention towards the environment. The first one (focused attention on the task at hand) is often discussed as selective attention, that is, a process of separating task-relevant and task-irrelevant stimuli. Selective attention is determined by current action goals (e.g., Allport, 1987; Folk et al., 1992; Frings & Wentura, 2006; Mast et al., 2014; Neumann, 1990). In contrast, the second attention process (attention towards the environment) is usually discussed in relation to screening the environment for possible dangers or chances irrespective of the current action goals, potentially in the service of survival (Öhman et al., 2001; Öhman & Mineka, 2001; Pratto & John, 1991; Wentura et al., 2000). Possibly, effects of self-relevance and emotional valence impact differently upon attention depending on these two functions of attention. Going back to our current study, in the emotional Stroop task, the content of the words cannot be related to the current action goal of naming the color. In the matching paradigm, on the contrary, we see prioritized processing of self-relevant stimuli (but not emotional stimuli) in a context where these stimuli are part of the current action goals. Although it might not always be easy to define in a particular paradigm whether a self-relevant or emotional stimulus is part of the environment or part of the current action goal, we think that many published effects of self-relevance or emotional valence should be re-evaluated with this question in mind. In particular, because in many paradigms it could not be clearly stated whether the attention-grabbing potential of a stimulus is due to its relatedness to current action goals (and hence the effect has to be discussed in light of the selective-attention function) or whether it is due to its relatedness to survival (and hence has to be discussed in light of the vigilance function of attention), it can be an exciting road for future research to further pursue this idea of an interaction between different stimulus dimensions with different attentional functions.

In sum, our results support the assumption of a specific effect of self-relevance on the way we process stimuli beyond its attention-grabbing characteristic. Self-relevance influences cognitive processing by privileging particular to-be-formed associations, which should be prioritized in future.

## Appendix 1

The overall 2 (relevance condition: *self-relevant* vs. *negative valence*) × 3 (shape: *relevant-associated* vs. *neutral1-associated* vs. *neutral2-associated*) × 2 (matching condition: *matching* vs. *non-matching*) × 2 (SOA: *short* vs. *long*) mixed-measures MANOVA with mean RTs as the dependent variable revealed that all main effects were significant, except the main effect of the between factor “relevance condition,” which missed the conventional criterion of significance, *F*(1, 36) = 3.43, *p* = .072, η_p_^2^ = .09. In detail, responses were significantly faster at long SOA, *F*(1, 36) = 433.94, *p* < .001, η_p_^2^ = .93, in matching trials, *F*(1, 36) = 46.87, *p* < .001, η_p_^2^ = .57, as well as for the relevant-associated condition (compared to one of the neutral-associated condition), *F*(2, 35) = 11.32, *p* < .001, η_p_^2^ = .39. Moreover, the effect of the association depended on the relevance condition, *F*(2, 35) = 5.24, *p* = .010, η_p_^2^ = .23, and this interaction again was affected by SOA as indicated by the significant three-way interaction of these three factors, *F*(2, 35) = 3.39, *p* = .045, η_p_^2^ = .16. No other interaction reached significance, all *F*s < 2.12, all *p*s > .148.

## Appendix 2

Mean RTs and error rates in the first task (Task 1, tone-discrimination task) are presented in Table A1. We analyzed these data to control for the precondition of the PRP paradigm that Task 1 is prioritized. In this regard, in the ideal case, Task 1 responses should not be differentially affected by the manipulations of Task 2. Hence, of special interest are effects involving the factor “SOA.” An overall 2 (relevance condition: *self-relevant* vs. *negative valence*) × 3 (shape: *relevant-associated* vs. *neutral1-associated* vs. *neutral2-associated*) × 2 (matching condition: *matching* vs. *non-matching*) × 2 (SOA: *short* vs. *long*) mixed-measures MANOVA with mean RTs in Task 1 as the dependent variable revealed a significant interaction of shape and SOA, *F*(2, 35) = 3.67, *p* = .036, η_p_^2^ = .17, as well as a significant interaction of shape, SOA, and relevance condition, *F*(2, 35) = 5.17, *p* = .011, η_p_^2^ = .23. The main effects for the factors match and relevance condition just missed the conventional criterion for significance, *F*(1, 36) = 3.46, *p* = .071, η_p_^2^ = .09 for match and *F*(1, 36) = 4.02, *p* = .053, η_p_^2^ = .10 for relevance condition. No further effects were significant, all *F*s < 2.26, all *p*s > .119.

To follow-up with the significant triple interaction, we conducted 3 (shape: *relevant-associated* vs. *neutral1-associated* vs. *neutral2-associated*) × 2 (SOA: *short* vs. *long*) MANOVAs for repeated measures (with matching conditions collapsed), separately for self-relevance and negative valence. For self-relevance, the two main effects were non-significant, *F*s < 1. That is, overall responses are at the same level in the short and long SOA condition. There was, however, a significant interaction, *F*(2, 18) = 5.97, *p* = .010, η_p_^2^ = .40, which was exclusively due to the contrast self-associated shape versus neutral shapes, *F*(1, 19) = 8.53, *p* = .009, η_p_^2^ = .31 (*F* < 1 for the second contrast). In detail, in the self-shape condition, responses to the tone were *M* = 57 ms (*SE* = 66 ms) faster in the short SOA condition compared to the long SOA condition; for the (collapsed) neutral conditions the corresponding difference was *M* = 6 ms (*SE* = 66 ms). Note that both differences were not significantly above zero (both *t*s < 1). Given this and the clearly non-significant main effect of SOA, we can conclude that, by and large, participants behaved as instructed: the tone task is prioritized so that RTs are at a comparable level in short and long SOA conditions.

For the negative-valence condition, the same 3 (shape) × 2 (SOA) MANOVA for repeated measures yielded non-significant effects, *F*(1, 17) = 2.42, *p* = .138, η_p_^2^ = .13 for the main effect of SOA, *F*(2, 16) = 2.72, *p* = .138, η_p_^2^ = .13 for the SOA × shape interaction (*F* < 1 for the main effect of shape). However, although the main effect of SOA was not significant, we should note that responses are on average *M* = 78 ms (*SE* = 50 m) *slower* in the short SOA condition compared to the large SOA condition. It might be of interest that the main effect test for SOA was burdened by an outlier (i.e., a participant who slowed down their Task 1 responses in the *long* SOA condition by 470 ms). To adequately account for this, we tested the average slowing in a t-test for trimmed means (see Wilcox, 1997) with a trimming of γ = .20; it yielded *t*(9) = 2.47, *p* = .036. Thus, with some caution we can conclude that in the negative condition, participants did not prioritize Task 1 to full extent.

The comparable analysis with error rates revealed a significant main effect of SOA, *F*(1, 36) = 74.53, *p* < .001, η_p_^2^ = .67, and the interaction of shape and relevance condition just missed the conventional criterion for significance, *F*(2, 35) = 2.71, *p* = .081, η_p_^2^ = .14. No further effects were significant, all *F*s < 2.31, all *p*s > .137.

**Table 4:** Mean RTs in milliseconds) and error rates (in %) in the first task of the psychological-refractory-period (PRP)-paradigm as a function of matching condition (*matching* vs. *non-matching*), relevance condition (*self-relevant* vs. *negative valence*), shape (*relevant-associated* vs. *neutral1-associated* vs. *neutral2-associated*), and stimulus-onset asynchrony (SOA; *100 ms* vs. *1,000 ms*). Standard deviations are given in parentheses

			SOA			
			100 ms		1,000 ms	
Matching condition	Relevance condition	Shape	RTs	ERs	RTs	ERs
matching	self-relevant	relevant	994 (167)	9.8 (6.7)	1065 (344)	4.5 (4.6)
		neutral1	1054 (215)	8.5 (6.4)	1052 (363)	3.5 (3.1)
		neutral2	1048 (227)	8.1 (7.8)	1068 (379)	3.1 (3.4)
	negative valence	relevant	924 (187)	10.3 (8.4)	873 (256)	4.2 (6.1)
		neutral1	938 (223)	11.4 (10.7)	868 (260)	4.9 (7.0)
		neutral2	948 (220)	12.9 (11.9)	855 (255)	3.9 (4.9)
non-matching	self-relevant	relevant	1050 (201)	9.4 (6.1)	1093 (381)	3.9 (3.1)
		neutral1	1073 (233)	9.3 (7.6)	1066 (390)	3.6 (3.6)
		neutral2	1057 (236)	9.4 (6.6)	1069 (394)	3.5 (3.2)
	negative valence	relevant	952 (216)	10.3 (10.3)	864 (251)	3.9 (3.1)
		neutral1	936 (226)	10.8 (9.5)	883 (285)	5.8 (7.2)
		neutral2	948 (231)	11.1 (10.4)	835 (266)	5.1 (6.9)

## Footnotes

1 Note that, so far, word concreteness is the only linguistic feature that has been shown to cause a prioritization in the matching task (Wade & Vickery, 2017). To ensure that the relevant label is not the most concrete label in the task and thereby to avoid the possibility that potential prioritization effects could be explained by word concreteness and not by their inherent relevance, we used concrete labels as the neutral control labels.
